# Molecular and geographic evolutionary support for the essential role of *GIGANTEAa* in soybean domestication of flowering time

**DOI:** 10.1186/s12862-016-0653-9

**Published:** 2016-04-12

**Authors:** Yan Wang, Yongzhe Gu, Huihui Gao, Lijuan Qiu, Ruzhen Chang, Shouyi Chen, Chaoying He

**Affiliations:** State Key Laboratory of Systematic and Evolutionary Botany, Institute of Botany, Chinese Academy of Sciences, Nanxincun 20, Xiangshan 100093 Beijing, China; Graduate University, Chinese Academy of Sciences, Yuquan Road 19, 100049 Beijing, China; The National Key Facility for Crop Gene Resources and Genetic Improvement (NFCRI)/Key Lab of Germplasm Utilization (MOA), Institute of Crop Science, Chinese Academy of Agricultural Sciences, 100081 Beijing, China; National Key Laboratory of Plant Genomic, Institute of Genetics and Developmental Biology, Chinese Academy of sciences, 100101 Beijing, China

**Keywords:** Domestication, Evolution, Flowering time, *GIGANTEA*, Haplotype, Soybean

## Abstract

**Background:**

Flowering time is a domestication trait of *Glycine max* and varies in soybeans, yet, a gene for flowering time variation has not been associated with soybean domestication. *GIGANTEA* (*GI*) is a major gene involved in the control of flowering time in *Arabidopsis*, although three *GI* homologs complicate this model in the soybean genome.

**Results:**

In the present work, we revealed that the geographic evolution of the *GIGANTEAa* (*GIa*) haplotypes in *G. max* (*GmGIa*) and *Glycine soja* (*GsGIa*). Three *GIa* haplotypes (H1, H2, and H3) were found among cultivated soybeans and their wild relatives, yet an additional 44 diverse haplotypes were observed in wild soybeans. H1 had a premature stop codon in the 10^th^ exon, whereas the other haplotypes encoded full-length GIa protein isoforms. In both wild-type and cultivated soybeans, H2 was present in the Southern region of China, and H3 was restricted to areas near the Northeast region of China. H1 was genetically derived from H2, and it was dominant and widely distributed among cultivated soybeans, whereas in wild populations, the ortholog of this domesticated haplotype H1 was only found in Yellow River basin with a low frequency. Moreover, this mutated *GIa* haplotype significantly correlated with early flowering. We further determined that the differences in gene expression of the three *GmGIa* haplotypes were not correlated to flowering time variations in cultivated soybeans. However, only the truncated *GmGIa* H1 could partially rescue *gi-2* A*rabidopsis* from delayed flowering in transgenic plants, whereas both *GmGIa* H2 and H3 haplotypes could significantly repress flowering in transgenic *Arabidopsis* with a wild-type background.

**Conclusions:**

Thus, *GmGIa* haplotype diversification may have contributed to flowering time adaptation that facilitated the radiation of domesticated soybeans. In light of the evolution of the *GIa* gene, soybean domestication history for an early flowering phenotype is discussed.

**Electronic supplementary material:**

The online version of this article (doi:10.1186/s12862-016-0653-9) contains supplementary material, which is available to authorized users.

## Background

The transition from vegetative to reproductive growth is an important developmental process in plants, and flowering time is controlled by the merger of complex networks including the photoperiod, vernalization, gibberellin, autonomy, and age pathway [1, 2]. These regulatory networks respond to endogenous cues and the external environment to maximize reproduction, thus flowering time is also an important agronomic trait in crop plants. These organisms constantly monitor environmental signals such as photoperiod, in particular, which is a primary signal, to adjust the timing of the floral transition. In *Arabidopsis*, the photoperiodic flowering pathway mainly comprises the *GIGANTEA* (*GI*), *CONSTANS* (*CO*), and *FLOWERING LOCUS T* (*FT*) genes, and this *GI*-*CO*-*FT* model is conserved in many plants [3–6]. The GI protein, unique to plants, is a nuclear protein that acts upstream of the photoperiod pathway at a junction between the circadian-clock and the flowering time pathway [6, 7]. GI can then induce *CO* and *FT* expression by an interaction with the Flavin-Binding, Kelch Repeat, F-Box 1 (FKF1) protein in a *CO*-dependent manner [8]. In addition, GI can directly or indirectly regulate *FT* expression in *CO*-independent manner by binding to *FT* promoter regions or by interacting with *FT* repressors [9, 10].

Flowering time is a domestication trait in various crops to which many genes have been attributed and characterized. The *vernalization* (*Vrn*) and *photoperiod* (*Ppd*) genes participated in the domestication and adaptation of wheat and barley [11]. *Heading date1* (*Hd1*), an ortholog of *Arabidopsis CONSTANS*, possibly underwent human selection to diversify the flowering time of rice during domestication or in early cultivation [12]. Additionally, the *FLOWERING LOCUS T*/*TERMINAL FLOWER 1* (*FT*/*TFL1*) gene family underwent selective sweeps during the evolution of cultivated sunflower (*Helianthus annuus*) [13]. *ZmCCT* (*Zea mays* CCT domain-containing protein) is involved in photoperiod sensitivity and could accelerate the spread of maize post-domestication [14]. *GI* homologs have been characterized with functional diversification in photoperiod flowering in several crops. It acts as a floral activator in *Pisum sativum* (*LATE BLOOMER1*), *Triticum aestivum* (*TaGI1*) and *Hordeum vulgare* (*HvGI*) [15–17], whereas it functions as a floral repressor in *Oryza sativa* (*OsGI*) and soybean (*GmGI*) [18–20].

Soybean is an important source of protein and edible oil for humans. Cultivated soybean (*Glycine max*) is thought to have been domesticated from wild soybean (*Glycine soja*), which was distributed in China as early as 5000–9000 years ago [21–23]. During the process of soybean domestication many phenotypic changes were observed in the seed size, shattering, flowering time, growth habit and plant architecture, leading to high grain yield and wide cultivation [24, 25]. Using genetic populations derived from crosses between wild and cultivated soybeans, potential target loci have been connected to soybean domestication [26]. Soybean whole genome sequencing further suggested that many genes are involved in soybean domestication [27–30]. However, only three domesticated genes have been functionally characterized and are involved in pod shattering, seed hardness, and determinate growth in soybeans [31–33].

Both wild and cultivated soybeans are generally short-day plants and are sensitive to photoperiods, although a few photoperiod-insensitive accessions were isolated in cultivars [34]. Moreover, cultivated soybeans usually flower earlier than their wild counterparts [24–26], indicating that early flowering is favored during soybean domestication, yet the genetic variation causing this difference are poorly known. Eight early maturity (*E*) loci, designed as *E1* to *E8*, have been demonstrated to be involved in soybean maturity [35]. *E1*, *E2*, *E3*, and *E4* are involved in soybean adaptation to different latitudes [35, 36]. Of which, *E2* encodes a GI homolog, a putative floral repressor [20]. The soybean genome contains three *GI* homologs [37], but only *GIa* plays a role in maturity and flowering in soybean [20], and interestingly appears to be under selection [30]. Whether this genetic variation of *GIa* is involved in the soybean domesticated process is unknown. With the aim to detect the possible role of *GI* in soybean domestication of flowering time, we focused on the variation of *GIa* alleles in wild and domesticated soybeans with a wide geographic distribution. We also investigated variations in *GIa* expression in soybeans with significant variations in flowering time. Because soybean transformation is extremely difficult, the role of *GmGIa* alleles in flowering time was further examined in transgenic *Arabidopsis*. Our analyses suggest that the molecular and functional evolution of *GmGIa* haplotypes demonstrate evidence for the selection of this gene in soybean flowering adaptation, and reinforce the hypothesis that the Yellow River region is likely the main origin of soybean domestication in China.

## Methods

### Plant materials and growth conditions

The *G. soja* and *G. max* populations were described [38] and the details are available in Dataset S1 (Additional file 1). These accessions were deposited in the Chinese National Soybean GeneBank (CNSGB) [38]. They were grown in the same field under natural light conditions (14.20 ± 0.79 h light/day) from May to September (Institute of Botany, Beijing, latitude 39.9, longitude 116.3) for recording flowering time. Three to five plants from each accession were planted, and the time from germination to the appearance of the first flower bud in each plant was recorded as flowering time. Five to three individuals of randomly selected soybeans were also grown in a greenhouse under short-day conditions (SD, 14 h darkness/10 h light at 25-27 °C) for gene expression studies. The cotyledons upon germination were set as 0 day (DAG0), and then the trifoliate leaves were harvested every 5 days till to 40 days in the two accessions ZYD03294 and ZDD22648. The trifoliate leaves at DAG15 were harvested in each accession of the soybean populations. To ease manipulation, each sampling was performed at the same time point everyday (4:00 pm) because *GI* orthologs are circadian clock-controlled genes in various plants [7, 37, 39–41]. Each biological sample composed of at least three individuals of each accession.

### Transgenic *Arabidopsis* analyses

The open reading frames (ORF) of *GmGIa* haplotypes were inserted into pCAMBIA1300 vector driven by cauliflower mosaic virus (CaMV) 35S promoter. The constructs were introduced into wild type *Arabidopsis thaliana* (Col) and its *gi-2* mutants mediated by GV3101 *Agrobacterium* using floral dipping [42]. The transgenic Arabidopsis plants were confirmed by RT-PCR. *Arabidopsis thaliana* plants were grown in a growth chamber under long-day conditions (16 h light/8 h darkness at 23-25 °C). The number of rosette leaves at bolting was recorded for the flowering time.

### Gene expression analysis

Total RNA of trifoliate leaves from soybeans was isolated using SV Total RNA Isolation System (Promega, USA). The complementary DNA (cDNA) was synthesized with the oligo (dT)_18_ primers following the instructions of the M-MLV cDNA synthesis kit (Invitrogen, USA). Quantitative RT-PCR (qRT-PCR) analysis was performed on an Mx3000P QPCR system (Stratagene, Germany) using SYBR Premix Ex Taq (TaKaRa, Japan). The soybean *Actin* (Glyma18g52780) was used as an internal control.

### Yeast two-hybrid assay

Protein-protein interaction was detected using the yeast two-hybrid system (Clontech, USA). The ORFs of *GmFKF1*, *GmGIa* alleles, *AtGI*, and *AtFKF1* were introduced into pGBKT7 and pGADT7 respectively. Interaction strength was quantified using the o-nitrophenyl-β-D-galactoside (ONPG) method (Clontech).

### Sequencing analyses

Genomic DNA was extracted from leaf tissues using Plant genome Kit (Tiangen, China). Six regions (A, B, C, D, E, and F) of *GIa* (Glyma10g36600) were sequenced and the amplicon length ranged from 423 to 750 bp, with the exception of *GIa*-E, which was 67 bp in length (Additional file 2: Figure S1). *GIa*-E was located in the 10^th^ exon and *GIa*-C comprised of partial intron 5, exon 6, and partial intron 6, whereas the others were evenly distributed among introns, with an interval space of around 5 kb. *GIa* haplotypes were identified using the concatenated sequences of these six sequenced fragments from each accession. For flanking sequence analysis, except for Glyma10g36680 that comprised partial intron 1, exon 2, and partial intron 2, the sequenced regions of the other four genes around *GIa* were located in non-coding regions, with interval spaces ranging from 40 kb to 90 kb and covering a 290-kb region on chromosome 10 (Additional file 2: Figure S1). For the control in nucleotide diversity analyses, a 647-bp genomic fragment (comprising partial intron 8, exon 9, intron 9, exon 10, and partial intron 10) for *GIb* (Glyma20g30980) on chromosome 20 and a 718-bp genomic fragment comprising partial exon 5 and partial intron 5 for *GIc* (Glyma.16G163200 in the latest version database as Gmax_275_v2.0, also Glyma09g07240 in Gmax_189_v 1.1 database) on chromosome 16 were included. These portions of the *GI* genes were amplified by PCR and sequenced using gene-specific primers. All DNA fragments were commercially sequenced in Taihe Biotechnology Company (Beijing, China). Primers used in the present work (Additional file 2: Table S1) were commercially synthesized in Taihe Biotechnology Company.

### Sequence alignments and phylogenetic analyses

The sequence alignments were performed using the ClustalX v1.81 program [43] with default parameters and alignments optimized via manual adjustments using BioEdit v7.0.5 [44]. Evaluation of nucleotide diversity (π) and nucleotide polymorphism (θ), and haplotype analysis were performed using DnaSP 5.10 software [45]. The neighbor-joining (NJ) trees were constructed using MEGA 5.0 [46] with bootstrap values for 1000 replicates. The median-joining haplotype network was constructed with Network 4.6 (Fluxus Technology). The geographic locations for different haplotypes in soybeans were mapped with DIVA-GIS version 7.5.0 (http://www.divagis.org).

### Statistical analyses

Besides sequencing analyses, each experiment/measurement was performed using three independent biological replicates or repeated three times unless stated otherwise. Related statistical analyses such as two-tailed student’s *t-*test for difference significance, one-way ANOVA test, Pearson correlation analysis and multiple regression analyses (linear model) were performed using SPSS 15.0.

## Results

### Variations in flowering time in soybean

**Fig. 1:**
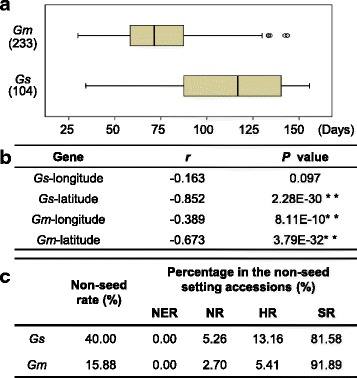
Flowering time variations in soybean. **a** Box-plot of flowering time variation in soybeans in Beijing. **b** Correlation of geographic origin (longitude, latitude) and Beijing flowering time in soybeans. *Gm*, *G. max*; *Gs*, *G. soja*. **c** The proportion of the Chinese accessions that did not produce seeds in Beijing. The collection covered different regions of China: NER, northeast region; NR, north region; HR, Huanghuai region; SR, south region. The numbers for accessions in different soybean populations are shown in brackets. The related details are presented in (Additional file 1: Dataset S1)

We first evaluated the variation of flowering time in soybean populations and correlated these phenotypes to geographic regions. Wild populations consisted of 104 individuals from China, Japan, and Korea. Domesticated accessions, distributed in different ecological regions in China, included 203 landraces and 30 cultivars (Additional file 1: Dataset S1). While these soybeans were grown in the same natural light conditions (see Methods), a significant difference in flowering time was observed between wild and cultivated soybeans (*P* = 4.98E-25). The flowering time for wild and domesticated soybeans was 111.02 ± 30.66 and 75.04 ± 24.88 days after germination (Fig. 1a) hinting that early flowering might be a breeding target for soybean domestication. Consistent with the previous findings [47], we also found that flowering time was negatively correlated with the geographic origin of the soybeans, particularly in the cultivated varieties (Fig. 1b). Forty-five accessions of wild (43.27 %) and 37 cultivated (15.88 %) varieties flowered, but did not produce seeds. Moreover, accessions, which set seeds flowered much earlier than those that failed to set seeds (Additional file 2: Table S2), and the proportion of these non-seed setting accessions was negatively correlated with their latitude of the collection site (*r* = -0.91, *P* = 2.09E-27; Fig. 1c), indicating that flowering time, affected by geographic latitude of planting place, is a barrier to the soybean radiation interception and wide cultivation. These results also suggest that flowering time is a key domestication trait for soybean fecundity.

### Sequence and expression diversity of *GI* homologs in soybean

**Fig. 2:**
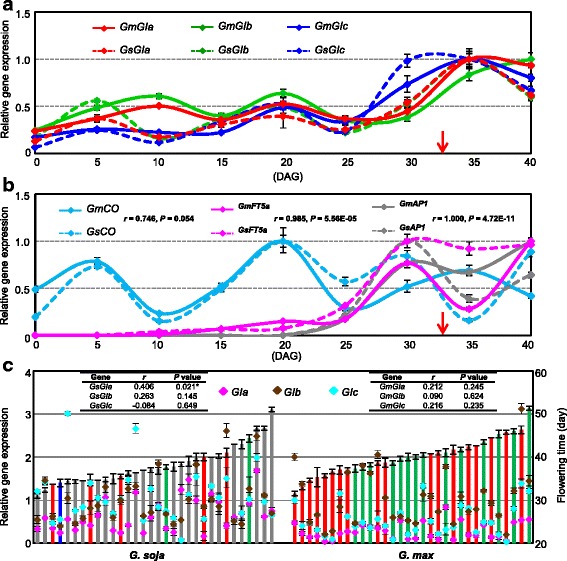
Expression of *GI* homologs and putative flowering time genes in soybeans. **a** The expression of *GIa*, *GIb*, and *GIc* during the seedling development of wild and domesticated soybeans under short-day conditions (SD). **b** The expression profiles of *CO*, *FT,* and *AP1* homologs during the seedling development of wild and domesticated soybeans under SD. DAG: days after germination. Flowering time is indicated by a red arrow. Both *Gs* (ZYD03294) and *Gm* (ZDD22648) flowered around DAG33. **c** The expression of *GIa*, *GIb*, and *GIc* in wild (32 accessions) and domesticated (32 accessions) populations (Additional file 1: Dataset S1) were investigated using qRT-PCR. Only *GIa* haplotypes were indicated as red, green, and blue for H1, H2, and H3, respectively. Others in wild soybeans are in gray. The flowering time (vertical columns) was recorded as the days from germination to the appearance of the first flower bud. The correlation coefficient between the flowering time and the *GI* expression in wild and domesticated populations is shown. *Gm*, *G. max*; *Gs*, *G. soja*

*GI* homologs exert a role in flowering time control and to better understand this role in soybeans, we evaluated their expression and sequence variation. Three *GI* homologs were found in soybean, and designated *GIa*, *GIb*, and *GIc* that were localized to chromosomes 10, 20, and 16, respectively (Additional file 2: Figure S2). We first examined the expression of *GI* homologs in one wild (ZYD03294) and one domesticate (ZDD22648) during its development into flowers. The two accessions had different flowering time under natural conditions (Additional file 1: Dataset S1). However, soybeans are strictly short-day plants. As control, the expression of the putative flowering time genes was therefore investigated during soybean development under short-day (SD) conditions. qRT-PCR analyses showed that all *GI* homologous genes had a similar dynamic expression profile (0.69 < *r* < 0.96, *P* < 0.038) in trifoliate leaves of wild and cultivated soybeans under SD, which was slightly elevated around 15 days after germination (DAG15) (Fig. 2a). The expression of the putative downstream genes of *GI*, i.e. *FT*, *APETALA1* (*AP1*), and *CO* orthologs were simultaneously investigated [1, 8–10], and we found that both *GmFT5a* and *GmAP1* expression started increasing around DAG15 and peaked after DAG30, while the *CO* homolog was constitutively expressed albeit its fluctuations during soybean development (Fig. 2b), thus each of these genes shared a similar expression profile during the development of these two accessions. However, *CO* expression was not correlated to the expression of *GmFT5a* and *GmAP1*. Concomitantly, the flowering time of the two soybean accessions was around DAG33 under SD, further indicating that wild and cultivated soybeans had different sensitivities to photoperiod. These observations in these two accessions seemed to support the GI-like regulating *FT* expression in the CO-independent manner in the control flowering time in soybean, but it could not tell whether *GI* homologs played a regulatory role. In addition, these observations suggested that DAG15 is an appropriate time for material harvest to evaluate gene expression diversity related to flowering in a soybean population level under SD.

To distinguish the role of the three *GI* homologs in flowering time, we harvested trifoliate leaves at DAG15 to expand the *GI* expression analysis to a larger soybean population. Thirty-two accessions of each of *G. soja* and *G. max* were randomly selected and were grown under SD conditions. All accessions flowered earlier under SD conditions, than seen in natural conditions (Additional file 1: Dataset S1), and flowering time was not significantly different between wild and domesticated populations under these conditions (Fig. 2c), for example, in natural light conditions, ZYD03294 and ZDD22648 flowered around DAG82 and DAG33, respectively, but they flowered around DAG33 under SD. These observations suggest that SD conditions circumvent the differential regulation of flowering time in soybean. However, the expression of *GIb* and *GIc* was not found to be correlated to flowering time variation in either population under SD conditions. Interestingly, *GsGIa* expression was correlated with flowering time (*r* = 0.41, *P* = 0.02) in wild populations, although this correlation was lacking for *GmGIa* in domesticated populations (Fig. 2c).

To evaluate the nucleotide diversity of soybean *GI* homologs, 33 landraces and 17 wild accessions were analyzed (Additional file 1: Dataset S1), which were randomly selected to maximally cover the geographic distribution. Sequencing analyses suggested that the *GIa*-E coding region covering the premature stop codon earlier identified by Watanabe et al. [20] showed a higher sequence diversity in cultivated soybeans than that in wild soybeans (Additional file 2: Table S3), which might be due to the extremely high frequency of this mutation in cultivated soybeans. Five genomic regions of *GIa* were further sequenced in these accessions (see Methods), and we observed that despite the high diversity in wild soybeans, relatively low diversity was detected in domesticated accessions (Additional file 2: Table S3). However, the sequence diversity of randomly selected *GIb* and *GIc* genomic regions was also evaluated (see Methods), and no significant difference was found between the wild and cultivated accessions (Additional file 2: Table S3). In all of these cases, the wild and cultivated soybeans shared a common set of haplotypes of each *GI* gene (Additional file 2: Table S3) hinting at a common ancestor. Phylogenetic trees constructed from the *GI* sequences showed that *GIa* was clearly distinguishable between the wild and the domesticated accessions; however, *GIb* and *GIc* were not, although they were clearly differentiated from *GIa* (Additional file 2: Figure S3). These observations imply that the specific selection on *GIa* may be responsible for the differences in flowering time between wild and cultivated soybean accessions, and thus perhaps the target of flowering time during soybean domestication.

### Selection of *GIa* alleles in soybean

**Fig. 3:**
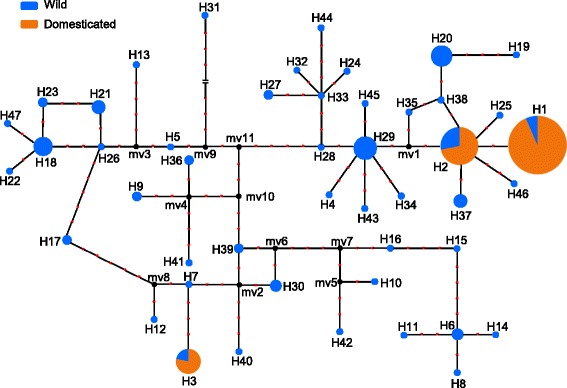
Median-joining haplotype networks of *GIa* in soybeans. To display the proportions of different germplasm inside each circle, 104 wild accessions were analyzed according to the frequency of different haplotypes in 203 landraces. The area of the circles is proportional to the frequency of each haplotype. Sections inside each circle reveal the proportion of different populations possessing the same haplotype. Black dots (“mv”) represent missing or extinct sequences. Red dots between two circles represent a mutational site

To substantiate the previous assumption, we investigated the allelic variation of *GIa* in wild and cultivated soybeans. Six polymorphic fragments of *GIa* were examined in all of the accessions (Additional file 1: Dataset S1; Additional file 2: Figure S4). Forty-seven haplotypes were found in the wild populations (104 accessions) designated H1–H47 based on the concatenated sequences of the six sequenced fragments, three of which (H1, H2, and H3) accounted for all accessions of the domesticated populations (Fig. 3). Median-joining haplotype networks showed that the genotypes could be divided into two branches: one included H1 and H2 haplotypes and the other branch covered the H3 haplotype. In the domesticated soybean populations of 233 accessions, H1 was the most frequent haplotype at 66.95 % followed by H2 at 21.89 % and H3 at 11.16 %, while the frequencies of these haplotypes were much lower in 104 accessions of wild soybeans at 4.81, 8.65 and 2.88 % respectively. Phylogenetic trees revealed that H3 might independently originate from H1 and H2, while the H1 radiated within cultivated soybeans (Fig. 3; Additional file 2: Figures S5–S7). Despite this, H1 was very closely related to H2, with the divergence being a single A/T transition that introduced a premature stop codon in the 10^th^ exon (Additional file 2: Figure S4), which was also characterized as *e2* [20].

**Fig. 4:**
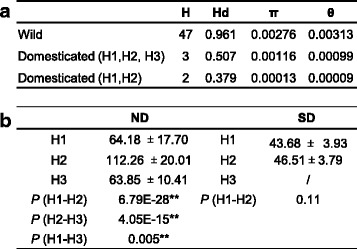
Soybean *GIa* is selected for flowering time variation. **a** The nucleotide diversity of *GIa* in soybeans. If H3 haplotype is identified as a wild allele, there are only two domesticated alleles. H: haplotypes, Hd: haplotype diversity, π: nucleotide diversity, θ: nucleotide polymorphism. **b** *GIa* haplotypes and soybean flowering time. *Gm*, *G. max*; *Gs*, *G. soja.* ND, natural-lighting conditions; SD, short-day conditions

The sequence diversity of the *GIa* gene was also estimated for all the haplotypes in wild and domesticated soybean populations in terms of nucleotide diversity (π) and polymorphism (θ) (Fig. 4a). The nucleotide diversity and nucleotide polymorphism of *GIa* was reduced in domesticates (*π* = 0.00013, *θ* = 0.00009) relative to wild populations (*π* = 0.00276, *θ* = 0.00313). While previous estimates show that domesticated soybeans retain 66 % (π) and 49 % (θ) of the nucleotide diversity of wild soybeans after the domestication bottleneck [21], both the π and θ of *GIa* were reduced to 4.7 and 2.9 % in domesticated accessions relative to wild accessions, indicating that *GIa* might have been under selection. A weak selection signal was detected in this particular gene locus [30]. However, the flanking down- and upstream sequences of the *GIa* locus on the chromosome 10 showed relatively high sequence diversity only when H3, a haplotype with the lowest frequency in cultivated soybeans, was excluded (Additional file 2: Figure S8), implying that *GmGIa* H3 had likely introgressed from a wild-type allele.

### Functional variations in domesticated *GIa* alleles that influence flowering time

In an attempt to infer the role of different *GIa* haplotypes in soybean flowering variation, phenotypic variation was attributed to each haplotype. In domesticated populations the earliest flowering varieties possessed H1 haplotype, while the latest flowering varieties had the H2 haplotype (Additional file 2: Figure S9a). The flowering time was significantly different between accessions possessing each haplotype of the cultivated accessions when employing both an ANOVA test (*P* < 0.05, Fig. 4b) and a multiple regression analysis (Additional file 2: Figure S9b). While the wild haplotypes were diverse, accessions possessing both H1 and H3 flowered relatively earlier than the accessions harboring H2 (Additional file 2: Figure S9a). These observations support the role of the diversity in *GmGIa* genotypes in the variation of soybean flowering time.

Sequencing revealed that the *GmGIa* haplotypes were putatively transcribed into three isoforms in the cultivated soybeans. Both H2 and H3 of *GmGIa* encoded a putative 1177-amino acid (AA) peptide, while H1 encoded a truncated isoform with 527 AA due to the premature stop codon in the 10^th^ exon (Additional file 2: Figure S10a). The H2 protein was distinguished from H3 isoform by only one conservative amino acid substitution (from V_220_ to I_220_), thus there may not be any of functional divergence between these two isoforms (Additional file 2: Figure S10a). However, H1 might be non-functional because it was prematurely terminated.

**Fig. 5:**
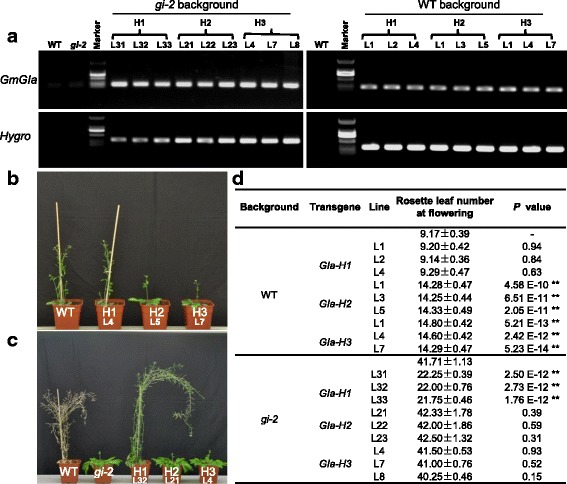
Analysis of *GmGIa* in transgenic *Arabidopsis.* **a** The expression of the transgenes in transgenic Arabidopsis plants*.* Both *GmGIa* and hygromycin resistance gene (*Hygro*) were detected using RT-PCR. The WT and *gi-2* were used as controls. **b** Overexpression of *GmGIa* in wild-type *Arabidopsis.* **c** Overexpression of *GmGIa* in *gi-2.* **d** Flowering time of transgenic plants. Rosette leaf number at bolting was recorded for flowering time. Difference relative to the wild-type or *gi-2* was evaluated using the two-tailed student’s *t*-test. The * indicates at *P* < 0.05 level, and the ** indicate at *P* < 0.01 level

GI forms a complex with the Flavin-Binding, Kelch Repeat, F-Box 1 (FKF1) protein to function in the photoperiod flowering pathway in Arabidopsis and this GI-FKF1 interaction is conserved in soybean [37]. In corroboration with our assumptions on the functional divergence of different GmGIa isoforms, using yeast-two hybrid assays we found that the H1 isoform had a weaker interaction with GmFKF1, while both H2 and H3 had a strong interactions with GmFKF1 (Additional file 2: Figure S10b). Next, we investigated the consequence of these differential interaction strengths in transgenic Arabidopsis. Three transgenic *Arabidopsis* lines of each genetic manipulation were verified by the expression of transgenes (Fig. 5a). As we expected, we found that in a wild-type (WT) Columbia background, H2 and H3 delayed flowering with a similar extent but H1 did not affect flowering (Fig. 5b–d). However, in the Arabidopsis *gi-2* background, a mutant with extremely late flowering, H2 and H3 transgenic plants did not rescue flowering time, whereas, surprisingly, H1 transgenic Arabidopsis lines partially rescued the floral phenotypic variation due to the *gi* mutation (Fig. 5b–d). In this scenario, GmGIa and AtGI may share the interacting protein (AtFKF1) in WT Arabidopsis, while GmGIa may solely occupy AtFKF1in the *gi-2* background. Indeed GmGIa isoforms differentially interacted with AtFKF1 in yeast (Additional file 2: Figure S10b). These results suggest that H2 and H3 haplotypes may repress flowering, in contrast to H1 possibly acting as an activator, thus increasing the frequency of H1 during domestication as a target for earlier flowering.

### Geographic distribution of soybean *GIa* haplotypes

**Fig. 6:**
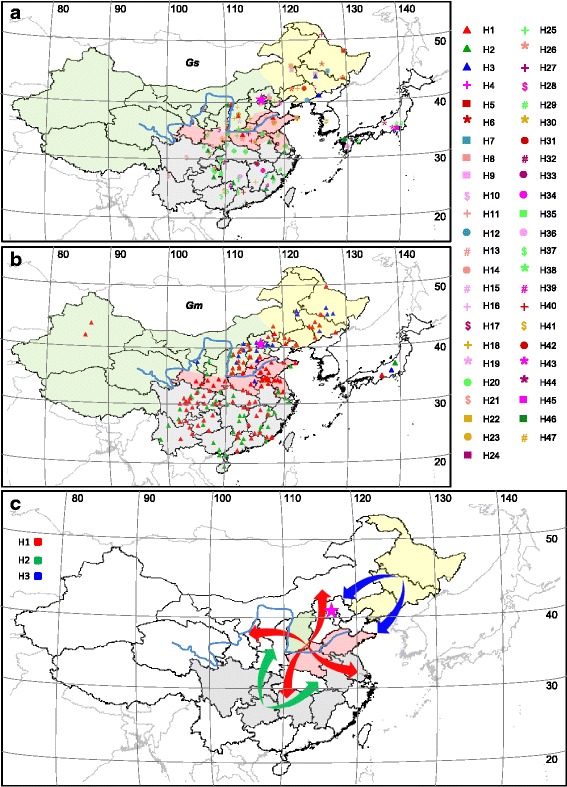
Geographic distribution of *GIa* haplotypes in soybeans. **a** Distribution of the *GsGIa* haplotypes of wild soybeans. 47 haplotypes were named as H1 to H47. **b** Distribution of the *GmGIa* haplotypes in soybean landraces. Three haplotypes were designated as H1, H2, and H3, which are shared by the wild soybeans. The haplotypes of domesticated soybeans in Japan are also restricted to H1 to H3 for 20, 7, and 5 individuals respectively [35]. *Gm*, *G. max*; *Gs*, *G. soja.* The colored symbols represent the different *GIa* haplotypes as indicated. **c** The *GmGIa* radiation pathways during soybean domestication. In wild populations, H1, H2, and H3 were restricted to different ecological regions in China: H1 in the Yellow River region (pink and green), H2 in NR (gray), and H3 in NER (yellow). Three major radiation events and places of *GmGIa* H1 (*red*), H2 (*green*), and H3 (*blue*) are proposed by different color arrows. H3 is assumed to be later introgressed from the wild soybeans in Northeast region of China, thus the very likely origin place of the domesticated soybeans might be the Yellow River region and the SR of China*.* However, considering the H1 distribution in wild soybeans, and the dominant H1 frequency in cultivated soybeans, the major domestication event might occur in the Yellow River region of China. The yellow, green, red, and gray areas respectively indicate NER, NR, HR and SR ecological regions in China. The blue line represents the Yellow River. The pink star indicates Beijing. Latitudinal and longitudinal coordinates are shown. The baseline map was created by us using DIVA-GIS version 7.5.0

To further understand the role of *GIa* in soybean domestication, we pinpointed the collection origin of these haplotypes geographically (Additional file 1: Dataset S1; Fig. 6). In wild soybean populations (Fig. 6a), 47 *GsGIa* haplotypes had a complex and diverse geographic distribution; however, a significant and interesting pattern was apparent when considering only the putatively domesticated haplotypes. H1 was restricted to Yellow River region of China that includes a part of NR (north region of China) and HR (Huanghuai region of China), while H2 and H3 were respectively limited to SR (south region of China) and NER (northeast region of China) with a very low frequency (Additional file 2: Figure S11a). Moreover, the wild haplotypes closely related to H2 were mainly limited to SR, while the closely related wild haplotypes of H3 were distributed in NER. Only one H2 and one H3 in wild soybean were collected near the Yellow River region. In soybean landraces (Fig. 6b), H2 and H3 had a distribution that overlapped perfectly with its wild orthologs. In contrast, H1 was found in the whole of China with a high frequency in domesticated populations (Additional file 2: Figure S11b). As a rare haplotype in wild soybeans, H1 was only found in Yellow River region of China and was spread to all the other eco-regions of Chinese cultivated soybeans, suggesting that H1 might have undergone artificial selection and human aided dispersal during soybean domestication in China. Interestingly, the three *GmGIa* haplotypes were also found in Japanese cultivars [35], but *GsGIa* H1 was not found in Japanese wild soybeans suggesting that cultivated soybeans in Japan may have been introduced from China.

## Discussion

Domestication is a complex process that involves human selection and plant adaptation to different environments that is accompanied by morphological and phonological changes [48] that distinguish cultivated crops from their progenitors [49]. Seed shattering traits in rice, changes of plant architecture in maize, fruit size in tomato, and flowering time in barley and wheat are domestication traits that have been described [11, 48, 50]. The evolution of flowering time is critical for plant domestication and the adaptation to new environments. As a domestication trait, flowering has been characterized in crop domestication, i.e. *Vrn* and *Ppd* genes in wheat and barley [11, 51], *Hd1* in rice [12], the *FT*/*TFL1* gene family in cultivated sunflower [13], and *ZmCCT* in maize [14]. To gain a greater understanding of soybean domestication, in the present work, we evaluated flowering time variation in soybean, and the evolution of the *GI* family, a key regulator of flowering time.

### Flowering time is a domesticated trait of soybean

Days-to-flowering is a domestication trait of soybean that differentiates cultivated accessions from their wild relatives [25]. Cultivated soybeans were domesticated to flower earlier than wild soybeans for high grain yield and wide cultivation [23]. Although a few soybean cultivars that have lost its photoperiod sensitivity were isolated [34], a reduction in photoperiod sensitivity is favored during soybean domestication. Both wild and cultivated soybeans are generally short-day plants, and flowering is delayed under long-day conditions. Because we found that the phase transition of some soybean accessions was delayed in Beijing under natural lighting, and while they ultimately flowered, they did not produce seed. Moreover, the extent of these phenotypic variations indeed negatively correlated with the latitude of the collection places of these non-reproductive soybeans (Fig. 1). Our findings are in line with the previous observations [36, 47]. However, we observed that the soybean accessions that were non-reproductive in Beijing’s environments were sensitive to photoperiod and flowered early under short-day conditions. These observations indicate the existence of a geographic barrier to soybean radiation that can be solved by a change in photoperiods. Thus, flowering time is an important soybean domestication trait, yet studies investigating genes associated with this domesticated trait were lacking. *GI* homologs, important regulators in the photoperiod pathway, function as activators for flowering in many plants; such as, *Arabidopsis*, pea, wheat and barley [7, 15–17], while some can act as repressors of flowering such as rice, soybean, and petunia [18, 20, 39]. Soybean has 3 *GI* homologs (*GIa*, *GIb*, and *GIc*); however, through comparisons of gene expression and sequence diversity variation between the wild and cultivated soybeans we demonstrated that soybean *GIa* is specifically involved in the earlier flowering time associated with domestication.

### Variations in *GIa* haplotypes are responsible for the observed differences in flowering time in soybean

In wild soybeans, substantial amount and diversity of haplotypes were seen; however, their association with flowering time variation is not easily established. Nevertheless, the variation in the *GIa* expression seemed to play a pronounced role in the variation of the flowering time. A previous study suggested that almost 81 % of rare alleles in the wild soybean populations were purged by the soybean domestication bottleneck [21]. In line with the previous whole genome association analysis [30], our gene-focused analysis of *GIa*’s flanking sequence in soybean landraces also conditionally supports selection on the *GmGIa* locus. Furthermore, we found that more than 93 % (44/47) of the wild haplotypes of *GIa* were lost during soybean domestication and breeding, and only H1, H2, and H3 were maintained in domesticated accessions. While H1 produced a truncated protein, H2 and H3 produced nearly indistinguishable, full-length proteins. In line with this previous work [20], we found that H1 is prevalent in cultivated accessions, and the accessions harboring H1 (also *e2*) show significantly earlier flowering with statistical power. However, for the first time, we found 5 wild soybean accessions harbored H1 and they usually flowered earlier (87.20 ± 15.23 days) than the average flowering time in all wild accessions (111.02 ± 30.66 days). Thus, the appearance and radiation of H1 seemed to play a prominent role in soybean domestication.

As expected, we further found that in transgenic Arabidopsis both H2 and H3 could not compensate the flowering defect in *gi-2* mutants, but could delay flowering in wild type Arabidopsis*,* suggesting that the full length GmGIa isoforms repress flowering in transgenic Arabidopsis. Therefore, our work reinforces that soybean *GIa* is a floral repressor, and indicates that *Arabidopsis* and *Glycine* may share a common regulatory and interacting network associated with GI. H1 is a mutated allele of *GIa*, and we also observed that H1 indeed had little effect on flowering in wild type Arabidopsis. However, beyond our expectations, it is interesting that in transgenic Arabidopsis the H1 isoform could promote flowering in *gi-2* mutants, thus hinting that H1 might not be a null mutation. The observations in transgenic analyses could be partially explained by different interacting capabilities of different GmGIa isoforms with AtFKF1, but they could also reflect differences in interacting networks of GI orthologs that regulate flowering between *Arabidopsis* and *Glycine*. Alternatively, the interacting network of GI orthologs might be relatively conserved, although these play either as a floral activator or repressor in different plants [15–20, 39]. The opposite effect of GI orthologs in its own hosts might be due to its sequence variations, because a single amino acid substitution could sufficiently reverse the role of a flowering time gene [52]. It could also be possible that the truncated soybean GIa (H1 isoform) could promote flowering instead of non-functionalization. While further studies are needed to investigate these hypotheses, nevertheless, *GmGIa* is a key participant in the regulation of flowering time in soybeans.

H3 might have originated from the northeast region of China (NER) and was restricted to the Northern latitude, whereas the soybeans in these regions tend to flower earlier. However, H3 repressed flowering in transgenic *Arabidopsis*. The present study observed that H3-harboring soybeans genetically deviated from H2-derived soybeans; therefore, other *E* loci might function in early flowering in H3-harboring soybeans. The frequency of H3 was relatively low in both wild-type and cultivated soybeans, which also indicated that H3 might be an introgressed allele of wild soybeans from NER. When the H3 was excluded, our selection analyses supported the suggestion from the recent whole genome association analyses that showed the possibility of selection on the *GIa* locus [30]. H1 might be the major selected *GIa* allele during the domestication or the postdomestication radiation of cultivated soybeans. Interestingly, our further comparative studies between wild and domesticated soybeans suggest that selection acted differentially on *GIa*. In wild soybeans, selection in nature mainly acts on the *GsGIa* expression variation among these *GsGIa* haplotypes. However, selection under cultivated conditions is clearly associated with the variation of the coding region of *GmGI* haplotypes. Among the three domesticated haplotypes, H1 is the most successful for early flowering and may have facilitated the radiation of soybeans after domestication.

### Geographic radiation of *GmGIa* alleles reflects soybean domestication processes

The processes of domestication vary substantially among crop species. With a single domestication event, maize was domesticated from its wild progenitor (*teosinte*), distributed in highland Mexico [53]. However, barley and rice were domesticated from their wild ancestors by two domestication events [54, 55]. Cultivated soybeans were hypothesized to have been domesticated from wild soybeans in China, but controversy existed about the origin of the first cultivated soybeans [27, 38, 56, 57]. The NER (northeast region of China), SR (south region of China), and Yellow River region in China were assumed to be the origins of cultivated soybean. In the present work, the geographic evolution and distribution of *GIa* alleles sheds light on the soybean domestication process (Fig. 6c).

The previous structural analyses also suggest that the genetic subdivisions of the soybean populations used in the present work were well clustered by geographic location such as NER, NR, HR, and SR in China (Additional file 2: Figure S11a; [38]), thus the population is ideal for understanding the soybean domestication process. We found that H1 is a rare haplotype in wild soybeans restricted to the Yellow River region in China, yet it is highly abundant in many cultivated soybean accessions from all detected geographic subgroups, suggesting that soybean domestication may have occurred in the Yellow River region. H1 was not detected in wild soybeans from Japan and Korea in the present study, although it was identified in Japanese cultivated soybeans [35]. However, a more extensive sampling of Japanese and Korean wild soybean would be required to test the hypothesis that soybean domestication occurred in the Yellow River region of China with H1 haplotype subsequently spreading to Japan, Korea and the rest of the world [38, 57, 58]. Both H2 (from SR) and H3 (from NER) were likely introduced into the domesticated soybeans by introgression. The distribution of H2 and H3 haplotypes beyond its sites of origin was limited by their relatively later flowering, thus establishing the current geographic pattern that lacks H3 in SR and H2 in NER. This scenario seems to support the hypothesis that soybean was domesticated from its wild progenitor in Yellow River region of China [31, 38].

H1 was closely related to H2, and it is possible that H1 may have been derived from H2 due to a single mutation in the 10^th^ exon, which could make the original domestication site be SR, the origin of H2. This is in line with the hypothesis of the soybean origination in south China [57]. In this scenario, the premature stop mutation occurred in H2 thus generating H1 in the Yellow River region. H1 was more efficient in promoting flowering with a wider adaptation than H2, thus H1 was therefore presumably then selected by ancient soybean breeders and quickly distributed to different regions of China. During the radiation of H1 to NER, H3 may have been introduced into cultivated soybeans by wild-type allele introgression. As a result, H1 exists in domesticated soybeans with a high frequency and wide distribution, while H2 and H3 are restricted to the regions near to their origin, and have very low frequency in the cultivated soybeans in China. Based on the archaeological record [22], multiple origins of domesticated soybean cannot be excluded. Independent recruitment of H3, in NER of China, a *GIa* allele functionally diverged from H2-derived H1, might partially support this notion. This assumption contradicts the hypothesis of a single domestication event in soybean [30, 38, 57], and thus the soybean domestication process is still in debate. Conclusively revealing the origin of soybean domestication requires a combined investigation of the evolution of multiple key domesticated genes and human historical activities. The present study showed that the evolution of *GIa* alleles plays a role in soybean domestication of flowering time, and the origin and radiation of H1 may primarily reflect the origin of cultivated soybeans. The distribution and frequency of the H1 haplotype among wild and cultivated soybeans supports the concept that the Yellow River region is most likely the main origin of soybean cultivars.

## Conclusions

As a critical trait for reproduction and adaptation to different environments, domesticating flowering time was a crucial component of soybean domestication. The *GIa* H1 haplotype that harbors a premature stop codon is an allele for an early flowering prevalent in domesticated soybeans. The wild H1 haplotype originated in the Yellow River region and is restricted to this area. However, the soybean accessions harboring H1 did not always flower early indicating the complexity of the flowering control pathway. Nevertheless, in light of the evolution of *GIa* gene, human selection for an early flowering phenotype might have at least occurred in the Yellow River region during soybean domestication.

## Ethics

Not applicable.

## Consent to publish

Not applicable.

## Availability of supporting data

The dataset supporting the results of this article is available in additional file 1, and additional file 2. Sequence data described in this article can be found in GenBank (http://www.ncbi.nlm.nih.gov) under the accessions of KU557045-KU557244 and KU664850-KU665234. In addition, the sequence alignments and phylogenetic trees were deposited in TreeBASE (http://www.treebase.org/) under the submission number S18817 (http://purl.org/phylo/treebase/phylows/study/TB2:S18817).

## Additional files

Additional file 1: Dataset S1. Detailed information of soybeans used in the present work. (XLSX 38 kb) Additional file 2: Figure S1. The genomic location of the *GIa* gene. **Figure S2.** A phylogenetic tree of *GIGANTEA* (*GI*) homologs. **Figure S3.** Phylogenetic analyses using the *GI* sequences in soybeans. **Figure S4.** Sequence variation of 47 *GIa* haplotypes in soybeans. **Figure S5.** A NJ phylogenetic tree of *GmGIa* sequences. **Figure S6.** A NJ phylogenetic tree of *GsGIa* sequences. **Figure S7.** A NJ tree of *GIa* sequences in wild and domesticated soybeans. **Figure S8.** Relative nucleotide diversity of *Gm* to *Gs* in five noncoding sites around *GIa*. **Figure S9.** Flowering time variation and *GIa* haplotypes in soybeans. **Figure S10.** *GmGIa* is associated with floral pathways. **Figure S11.** Haplotype frequency of *GIa* in soybeans. **Table S1.** Primers used in the present study. **Table S2.** Flowering time and seed setting in soybean. **Table S3.** Nucleotide diversity of soybean *GI* homologs. (PDF 2065 kb)

## Abbreviations

cDNA: complementary DNA
*GI*: *GIGANTEA*
NJ: neighbor-joining
ONPG: o-nitrophenyl-β-D-galactoside
ORF: open reading frame
qRT-PCR: quantitative RT-PCR
RT-PCR: reverse transcription –polymerase chain reaction
SD: short-day condition
