# Supplementary material for: Molecular and geographic evolutionary support for the essential role of GIGANTEAa in soybean domestication of flowering time
Source: BMC Evol Biol. 2016 Apr 12;16:79. doi: 10.1186/s12862-016-0653-9 (PMC4830004; doi:10.1186/s12862-016-0653-9)
Supplement: Additional file 2: Figure S1. — The genomic location of the GIa gene. Figure S2. A phylogenetic tree of GIGANTEA (GI) homologs. Figure S3. Phylogenetic analyses using the GI sequences in soybeans. Figure S4. Sequence variation of 47 GIa haplotypes in soybeans. Figure S5. A NJ phylogenetic tree of GmGIa sequences. Figure S6. A NJ phylogenetic tree of GsGIa sequences. Figure S7. A NJ tree of GIa sequences in wild and domesticated soybeans. Figure S8. Relative nucleotide diversity of Gm to Gs in five noncoding sites around GIa. Figure S9. Flowering time variation and GIa haplotypes in soybeans. Figure S10. GmGIa is associated with floral pathways. Figure S11. Haplotype frequency of GIa in soybeans. Table S1. Primers used in the present study. Table S2. Flowering time and seed setting in soybean. Table S3. Nucleotide diversity of soybean GI homologs. (PDF 2065 kb) [file 12862_2016_653_MOESM2_ESM.pdf]

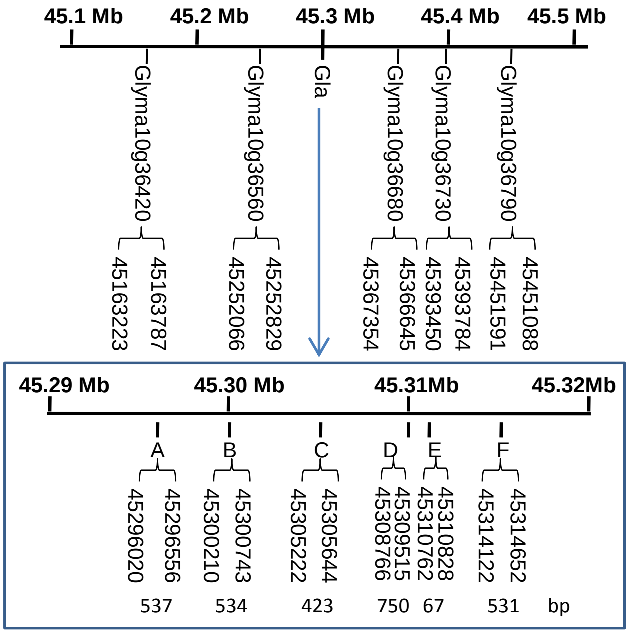

Figure S1. The genomic location of the *Gla* gene.
The six sequenced fragments of the soybean *Gla* gene are shown in the box. The numbers indicate the genomic location of genes on chromosome 10 using Williams 82 as a reference.

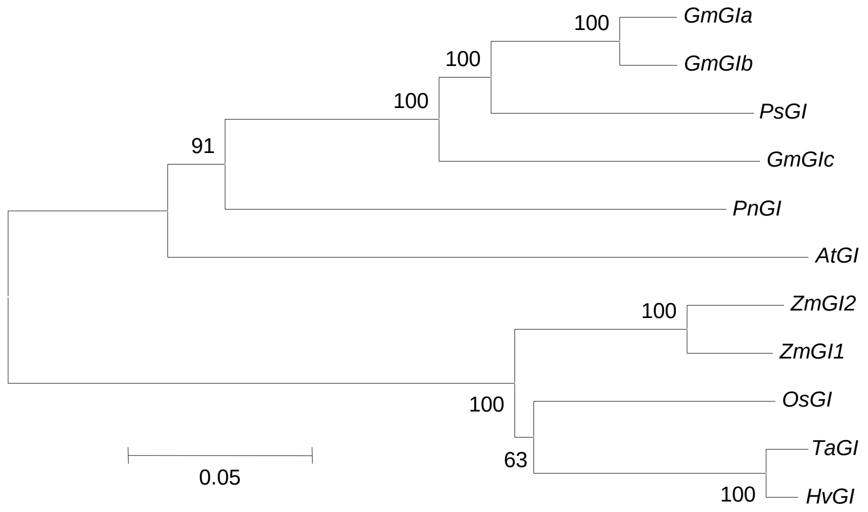

Figure S2. A phylogenetic tree of *GIGANTEA* (*GI*) homologs.
The genes included *GmGIa* (*Glycine max*, Glyma10g36600) on chromosome 10, *GmGIb* (*G. max*, Glyma20g30980) on chromosome 20, *GmGIc* (*G. max*, Glyma.16G163200 in the latest version database as Gmax_275_v2.0, also Glyma09g07240 in Gmax_189_v 1.1 database) on chromosome 16, *PsGI* (*Pisum sativum*, EF185297), *ZmGI1* (*Zea mays*, Zm2g10710), *ZmGI2* (*Z. mays*, Zm5g844173), *OsGI* (*Oryza sativa*, Loc_Os01g08700), *TaGI1* (*Triticum aestivum*, AF543844), *HvGI* (*Hordeum vulgare*, AY740523), *AtGI* (*Arabidopsis thaliana*, At1g22770), and *PnGI* (*Pharbitis nil*, AB265781). Bootstrap values for 1000 replicates are shown.

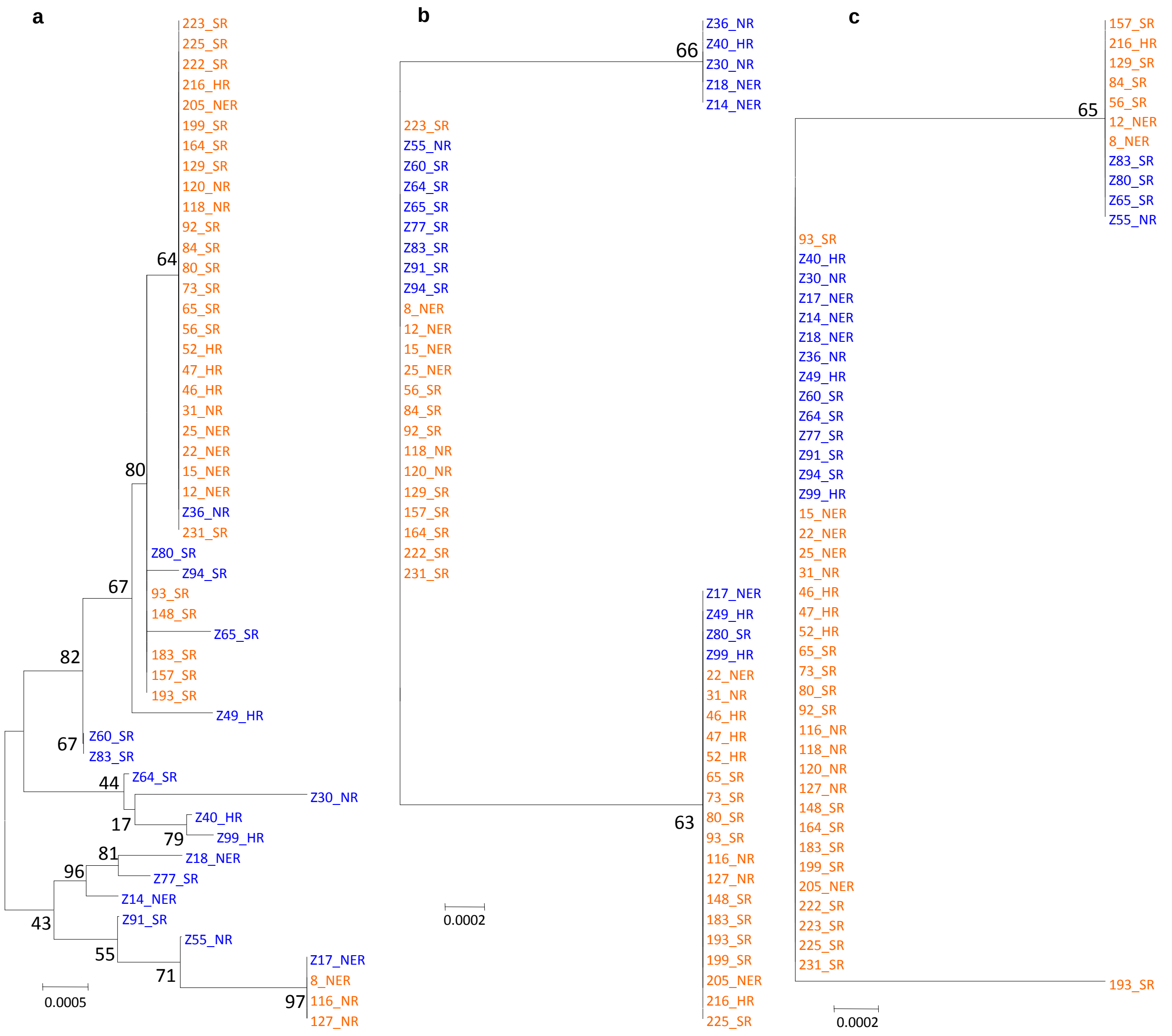

Figure S3. Phylogenetic analyses using the *GI* sequences in soybeans.
a-c The NJ trees of *GIa* (**a**), *GIb* (**b**), and *GIc* (**c**). Domesticated (orange) and wild (blue) populations consist of 33 and 17 individuals respectively. NER, northeast region of China; NR, north region of China; HR, Huanghuai region of China; SR, south region of China. Bootstrap values for 1000 replicates are shown.

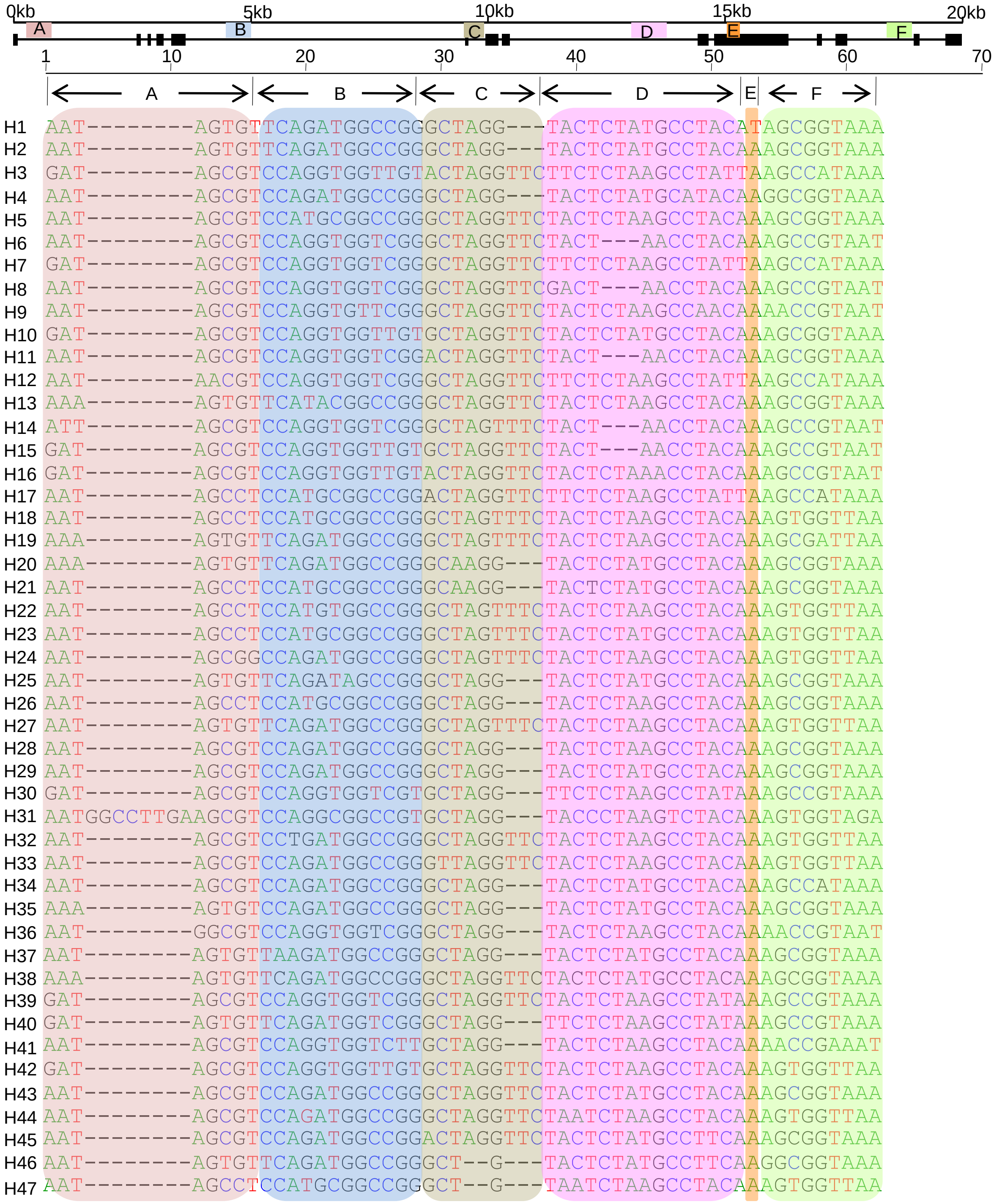

Figure S4. Sequence variation of 47 *GIa* haplotypes in soybeans.
The position of variable nucleotides among all sequenced sections in the referenced genomic structure of Williams 82 was given at the top of the picture. Sequence variation of the six analyzed fragments of *GIa* was labeled in the matrix of variable sequences of 47 kinds of haplotypes.

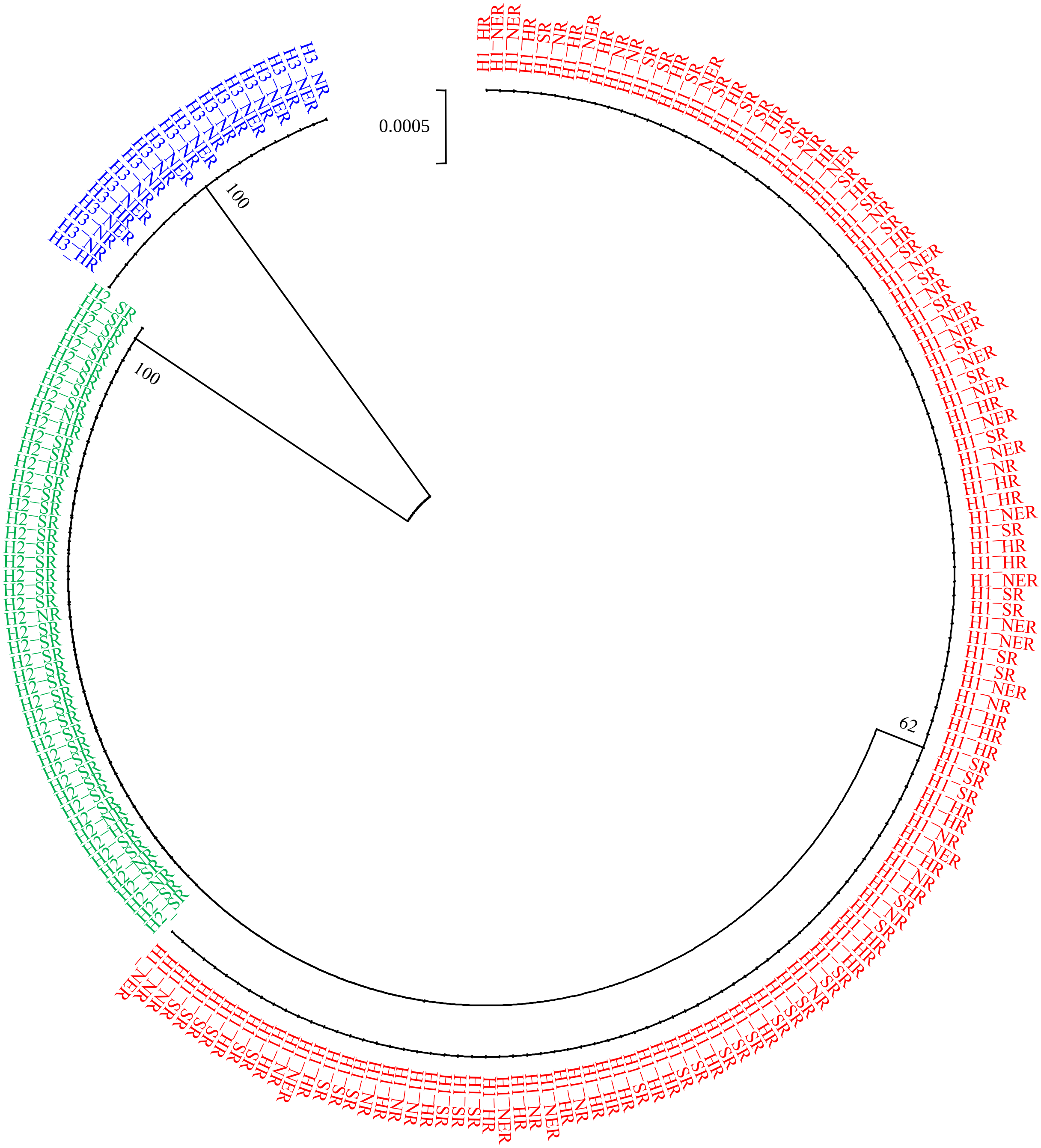

Figure S5. A NJ phylogenetic tree of *GmGla* sequences.
203 landraces from China were investigated. Red, green, and blue indicates accessions possessing H1, H2 and H3 haplotypes respectively. NER, NR, HR, SR, and bootstrap values for 1000 replicates are shown.

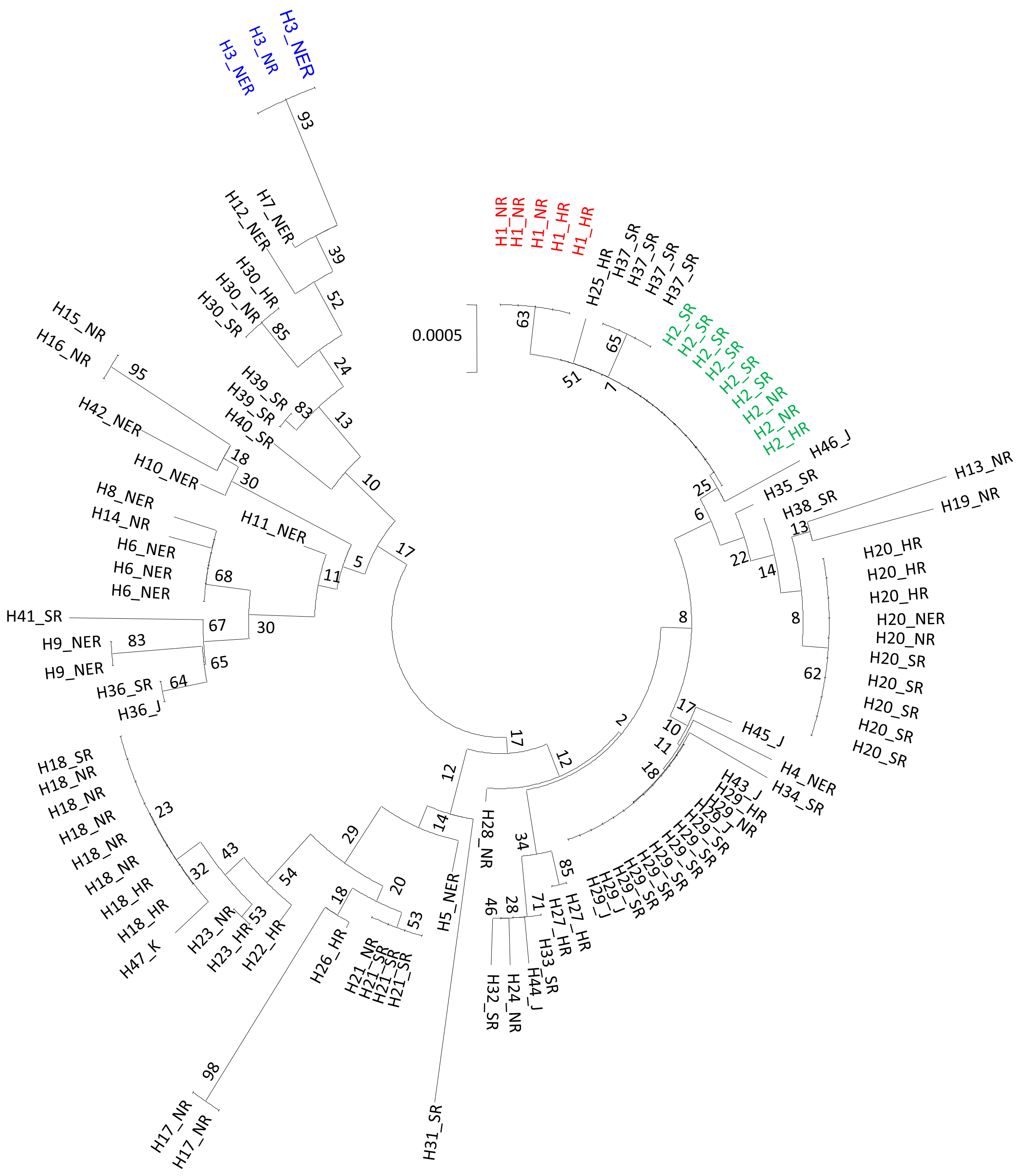

Figure S6. A NJ phylogenetic tree of *GsGla* sequences.
104 accessions of wild soybeans from China, Japan, and Korea were investigated. Red, green and blue indicates accessions possessing H1, H2, and H3 haplotypes respectively; black indicates others haplotypes in wild soybeans. K, Korea; J, Japan. NER, NR, HR, SR, and bootstrap values for 1000 replicates are shown.

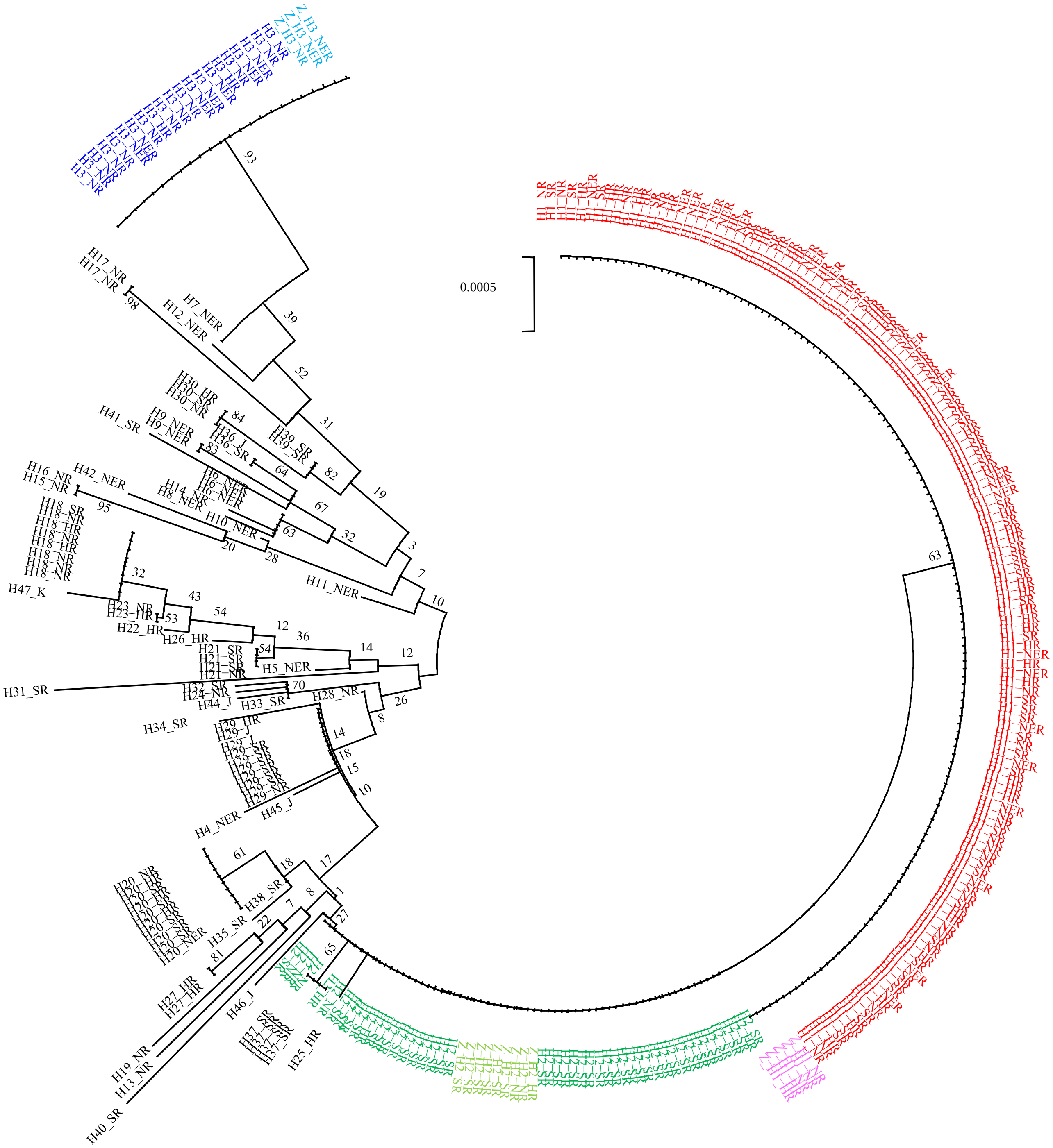

Figure S7. A NJ tree of *GIa* sequences in wild and domesticated soybeans.
Data are from Figure S5 and S6, thus the symbols and meanings are same as explained in these two figures. The haplotypes in black and the colorful ones beginning with a "Z" indicated the origin of wild-type soybeans.

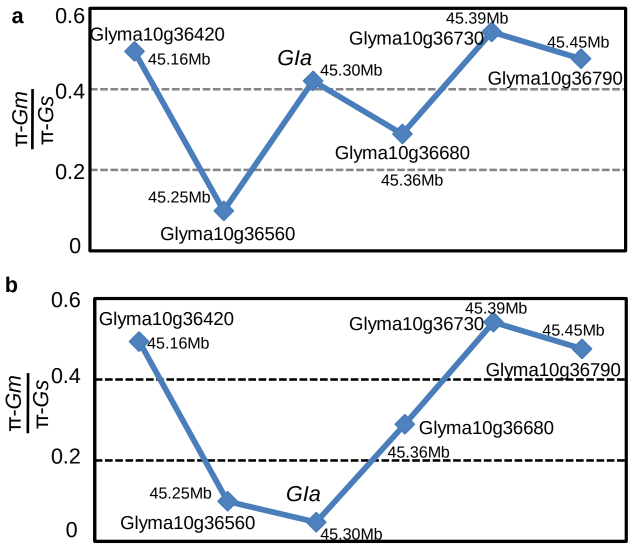

Figure S8. Relative nucleotide diversity of *Gm* to *Gs* in five noncoding sites around *Gla*.
a Nucleotide diversity when H1, H2, and H3 are considered.
b Nucleotide diversity when H3 is excluded.

a

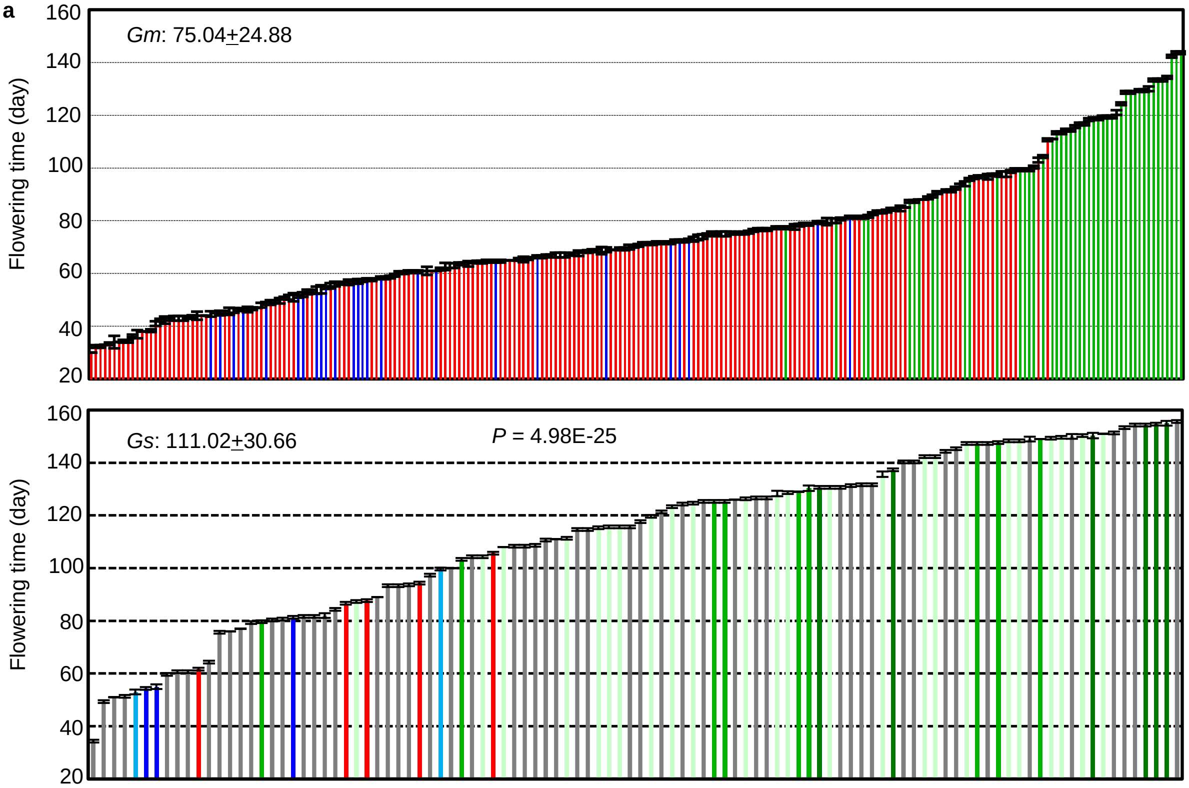

b

Factor	Unstandardized Coefficients	Standardized Coefficients	*t*	Significance
H2	106.659 ± 2.565	_	41.579	0.000
H1	-39.305 ± 2.955	-0.744	-13.302	0.000
H3	-47.492 ± 4.414	-0.602	-10.758	0.000

Figure S9. Flowering time variation and *GIa* haplotypes in soybeans.
a Flowering time variation in the domesticated (upper graph) and wild (lower graph) soybeans. Domesticated populations (233 accessions) possessed H1, H2, and H3, and wild populations (104 accessions) had 47 haplotypes designed H1 to H47. H1, H2, and H3 are set in red, green, and blue respectively. Haplotypes that are phylogenetically near to H2 and H3 are shown in colors similar to green and blue respectively. The other haplotypes were set in gray. *Gm*, *G. max*; *Gs*, *G. soja*. **b** The effect of different haplotypes on Beijing's flowering time was estimated by a multiple regression model. The flowering time of H2 was used as a control.

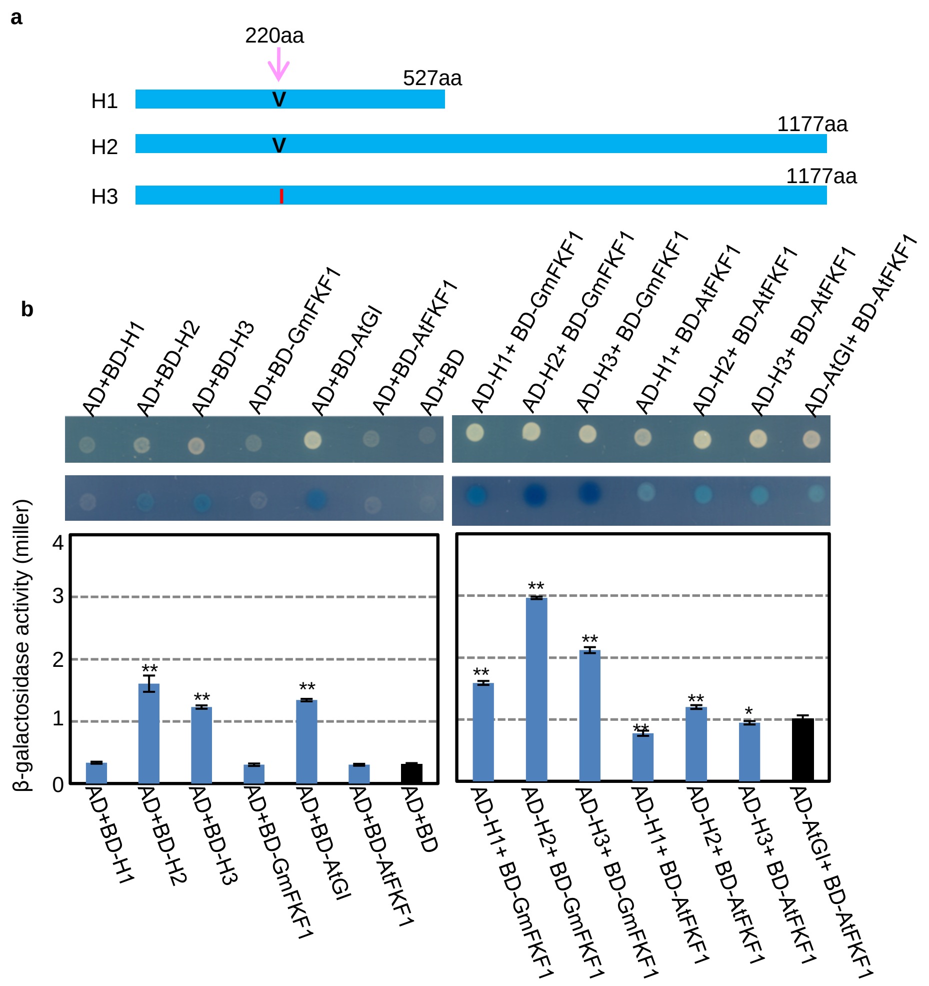

Figure S10. *GmGIa* is associated with floral pathways.
a Structures of GmGIa proteins. H1 isoform contains 527 aa due to the premature stop codon in the 10th exon. H2 and H3 isoforms have 1177 aa with one transition (V/I) as indicated. **b** Protein-protein interaction of FKF1 and different GI isoforms. The images above showed the growth of yeast cells in SD/-Trp/-Leu/-His/-Ade and β- galactosidase assay. Both cell growth and blue coloration indicate the interaction between the proteins as indicated. The graph below shows interaction strength. The interaction strength was quantified using the o-nitrophenyl-β-D-galactoside (ONPG) method. Significant difference relative to the combinations as indicated in black columns was evaluated using the two-tailed Student's *t*-test. The * indicates at $P < 0.05$ level, and the ** indicate at $P < 0.01$ level.

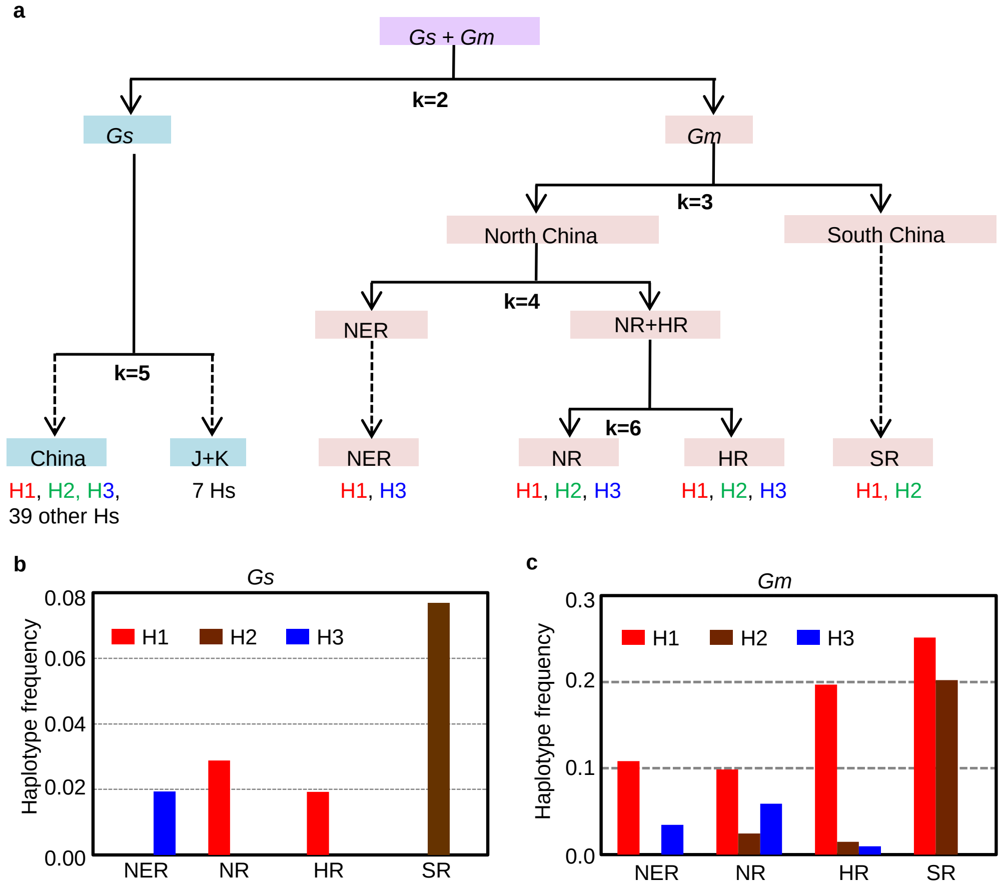

Figure S11. Haplotype frequency of *GIa* in soybeans.
a Soybean population structure was modified from the previous work [38]. The distribution of H1, H2, and H3 are shown in different subgroups. Hs, Haplotypes; NER, northeast region of China; NR, north region of China; HR, Huanghuai region of China; SR, south region of China; K, Korea; J, Japan. **b** The frequency of haplotypes in different geographic regions of China in wild soybeans. **c** The frequency of haplotypes in different geographic regions of China is displayed in domesticated soybeans. 104 accessions of wild soybeans and 203 landraces were included. *Gm*, *G. max*; *Gs*, *G. soja*.

Table S1. Primers used in the present study.

usages	gene	Primer	sequence
haplotype analysis	*GIa-A*	GI160F	GAGTAAAGCTTTTAAAACTTCATCAG
	GIa-A	GI600R	GCCACATATTGAGCTTAACAAACTTC
	GIa-B	I5-800F	CATTAGATCGAGCTACTGCATT
	GIa-B	I5-1610R	CCCTTTTTCATTAAGCATTACTCCC
	GIa-C	GI10600F	AATTATCTTGTTATGCCTTGAGCGC
	GIa-C	GI11320R	AAAAAGTCATTTCCTTATCATATTTC
	GIa-D	I8-F1	GTGAGTTAGTTAGTTAGTTAGAGGGAC
	GIa-D	I8-R1	CATATATTTAATAGGACCATCACTACCG
	GIa-E	GI10-1475F	GCAACCCCACTACAGCCTCCCGTG
	GIa-E	GI10-1700R	TTTTTTGAGGCAGAGCCAAAGCCT
	GIa-F	I11-F	GAAACCATGGTCAAATCAACAATAT
	GIa-F	I11-R	TGAAACACCAAATCCTCCCTTCACAGTCC
Expression analysis	*GIa*	GI-10F	GCTCTGCCTCAAAAAACCTGGCCG
	GIa	GI-10R	GAAGCTAAATGATTATTCCCCCG
	GIb	GI-20F	GCTCTGCCTCGAAAAACCTTGCCA
	GIb	GI-20R	GAAGCTAAACGATTATTCCCACA
	GIc	GI-09F	CAAAATATAGCCAAGCCTGTCAAAATAG
	GIc	GI-09R	GGGATGAATCCTCAGAAGTCGAAGT
	GmFT5a	FTF5	ATGAGAGCCCGAACCCTTCAGTA
	GmFT5a	FTR5	TCTCCTTCCACCGCAACCACGC
	GmAP1	APF4	AAACATCCGCACAAGGAGGAATGAT
	GmAP1	APR4	GCCTATGTTCAAACTGGTGCGAAGG
	GmCO	COF	GAGTGCTACTGTCCCTAACACCAA
	GmCO	COR	CCAGCGAAATGTTGGTGCTGAGC
	Actin	ActinF	ATCTTGACTGAGCGTGGTTATTCC
	Actin	ActinR	GCTGGTCCTGGCTGTCTCC
Constructs for transformation	*H2/H3*	GI10-3XbaIF	ATCTCTAGAATGTCGTCATCTTCGTCTTC
	H2/H3	GI10KpnIR	TATGGTACCCATGGAAATAGTACAGCCTAACTCT
	H1	GI10-3XbaIF	ATCTCTAGAATGTCGTCATCTTCGTCTTC
	H1	NGI10KpnIR	TATGGTACCCATAAGACATGCCTCTGATGG
Constructs for yeast two-hybrid assay	*H2/H3*	GI10-EcoRIF	TATGAATTCATGTCGTCATCTTCGTCTTCGATGGC
	H2/H3	GI10-3KSalIR	TACGTCGACTCACATGGAAATAGTACAGCC
	H1	GI10-EcoRIF	TATGAATTCATGTCGTCATCTTCGTCTTCGATGGC
	H1	GI10-3NSalIR	TACGTCGACTTACATAAGACATGCCTCTGATGG
	GmFKF1	FKF1-NcoIF	TATCCATGGCTGTGACAAAAGAGAAAGAGGATGTTCG
	GmFKF1	FKF1-EcoRIR	ATAGAATTCTCACAGGTCAGAGTCCTGTC
	AtGI	AtGI-ECORIF	TATCGAATTCATGGCTAGTTCATCTTCATCTG
	AtGI	AtGI-BamHIR	ATATGGATCCCTATTGGGACAAGGATATAGTACAG
	AtFKF1	AtFKF-NdeIF	ATATCATATGGCGAGAGAACATGCGATCGGA
	AtFKF1	AtFKF-SalIR	TATTGTCGACTTACAGATCCGAGTCTTGCCG
nucleotide diversity	*GmGIb*	20I8-F	AGAATAACTGTGATGCTGGCATTAATGG
	GmGIb	20I8-R	CGTGAGTGACTGTAAAAAATAAGAATAATC
	GmGIc	9I-6F	CATATACAAAATGGAAAGGCAATATTG
	GmGIc	9I-6R1	GGGTGATTTATGTTAGATAGGGAGA
	Glyma10g36420	36420F	CTGCAATGTCCTAACTTCTTTGGTAATGA
	Glyma10g36420	36420R	GGATAAGGAGAAGGTTACGAATGGGT
	Glyma10g36560	36560F	CCTGCACAAGTGAGGCTTCGACCTTCTT
	Glyma10g36560	36560R	GAAATGTTACAAGTTGCGTGATAAAATAC
	Glyma10g36680	36680F	CTGCATCTTTCTTTCTGTTTACAATATCA
	Glyma10g36680	36680R	GCAAAGGTTAGCCCGTCTCTC
	Glyma10g36730	36730PR	GGGAAATGAAAAGAAAAATTTGTGAGAGTT
	Glyma10g36730	36730PF	CACTCACACCCTTCTGTTTTTTA
	Glyma10g36790	36790PF	AGAGAGTTTAATCCAGACATGACAC
	Glyma10g36790	36790PR	TCACCACTCACCACCACCTGAATC

GmFT detect the expression for *GmFT5a* (Glyma16g04830); *GmCO* for Glyma18g51320; *GmAP1* for Glyma01g08150; GmFKF1 protein for Glyma05g34530.

Table S2. Flowering time and seed setting in soybean.

	Lines	Flowering time (days)
Gs	with seeds	92.55 ± 25.73
	without seeds	139.10 ± 12.35
Gm	with seeds	66.62 ± 16.05
	without seeds	119.68 ± 12.72

Table S3. Nucleotide diversity of soybean *GI* homologs.

	Gene	Haplotype	Hd	π	θ	π-*Gm* / π-*Gs*
GI homologs	*GIa-Gm*	3: H1-H3	0.373	0.00072	0.00131	0.2416
	GIa-Gs	16: H1-H16	0.993	0.00298	0.00313	
	GIb-Gm	2: H1, H2	0.504	0.00078	0.00038	0.6142
	GIb-Gs	3: H1, H2, H3	0.676	0.00127	0.00091	
	GIc-Gm	3: H1, H2, H3	0.398	0.00057	0.00067	0.9194
	GIc-Gs	2: H1, H2	0.442	0.00062	0.00039	
GIa	*GIa*-A-*Gm*	2: H1-H2	0.121	0.00045	0.00092	0.1765
	GIa-A-*Gs*	6: H1-H6	0.846	0.00255	0.00275	
	GIa-B-*Gm*	2: H1-H2	0.170	0.00113	0.00231	0.2173
	GIa-B-*Gs*	8: H1-H8	0.882	0.00520	0.00499	
	GIa-C-*Gm*	2: H1-H2	0.121	0.00029	0.00059	0.1973
	GIa-C-*Gs*	4: H1-H4	0.551	0.00147	0.00211	
	GIa-D-*Gm*	2: H1-H2	0.170	0.00065	0.00132	0.2902
	GIa-D-*Gs*	7: H1-H7	0.765	0.00224	0.00238	
	GIa-E-*Gm*	2: H1-H2	0.379	0.00565	0.00368	3.2102
	GIa-E-*Gs*	2: H1-H2	0.118	0.00176	0.00441	
	GIa-F-*Gm*	2: H1-H2	0.170	0.00046	0.00093	0.1299
	GIa-F-*Gs*	6: H1-H6	0.801	0.00354	0.00334	

Gm and *Gs* consist of 33 landraces and 17 wild individuals respectively, distributed in different ecological regions of China. *GIb* (Chromosome 20, 40759657 to 40759011); *GIc* (Chromosome 16, 32198084 to 32198801). The A, B, C, D and F part of *GIa* located on the chromosome 10 from 45296020 to 45296556, 45300210 to 45300731, 45305222 to 45305644, 45309515 to 45308766, 45310762 to 45310828, 45314330 to 45314652, respectively.
